# The use of bedside echocardiography in the care of critically ill patients - a joint consensus document of the *Associação de Medicina Intensiva Brasileira*, *Associação Brasileira de Medicina de Emergência* and *Sociedade Brasileira de Medicina Hospitalar*. Part 2 - Technical aspects

**DOI:** 10.5935/2965-2774.20230310-en

**Published:** 2023

**Authors:** José Augusto Santos Pellegrini, Ciro Leite Mendes, Paulo César Gottardo, Khalil Feitosa, Josiane França John, Ana Cláudia Tonelli de Oliveira, Alexandre Jorge de Andrade Negri, Ana Burigo Grumann, Dalton de Souza Barros, Fátima Elizabeth Fonseca de Oliveira Negri, Gérson Luiz de Macedo, Júlio Leal Bandeira Neves, Márcio da Silveira Rodrigues, Marcio Fernando Spagnól, Marcus Antonio Ferez, Ricardo Ávila Chalhub, Ricardo Luiz Cordioli

**Affiliations:** 1 Department of Intensive Care, Hospital de Clínicas de Porto Alegre, Universidade Federal do Rio Grande do Sul - Porto Alegre (RS), Brazil; 2 Department of Intensive Care, Hospital Universitário Lauro Wanderley - João Pessoa (PB), Brazil; 3 Department of Intensive Care, Hospital Nossa Senhora das Neves - João Pessoa (PB), Brazil; 4 Department of Emergency Medicine, Hospital Geral de Fortaleza - Fortaleza (CE), Brazil; 5 Universidade do Vale do Rio dos Sinos - São Leopoldo (RS), Brazil; 6 Department of Intensive Care, Hospital Nereu Ramos - Florianópolis (SC), Brazil; 7 Cardiovascular Intensive Care Unit, Hospital Cardiopulmonar Instituto D’Or - Salvador (BA), Brazil; 8 Intensive Care Unit, Hospital Universitário de Vassouras - Vassouras (RJ), Brazil; 9 Intensive Care Unit, Hospital Geral Roberto Santos - Salvador (BA), Brazil; 10 Department of Emergency, Hospital de Clínicas de Porto Alegre, Universidade Federal do Rio Grande do Sul - Porto Alegre (RS), Brazil; 11 Department of Hospital Medicine, Hospital Mãe de Deus - Porto Alegre (RS), Brazil; 12 Intensive Care Unit, Hospital Beneficência Portuguesa - Ribeirão Preto (SP), Brazil; 13 Department of Echocardiogram, Hospital Santo Antônio, Obras Sociais Irmã Dulce - Salvador (BA), Brazil; 14 Department of Intensive Care, Hospital Israelita Albert Einstein - São Paulo (SP), Brazil

**Keywords:** Echocardiography, Critical illness, Ventricular function, left, Ventricular function, right, Shock, Hemodynamics, Surveys and questionnaires

## Abstract

Echocardiography in critically ill patients has become essential in the evaluation of patients in different settings, such as the hospital. However, unlike for other matters related to the care of these patients, there are still no recommendations from national medical societies on the subject. The objective of this document was to organize and make available expert consensus opinions that may help to better incorporate echocardiography in the evaluation of critically ill patients. Thus, the *Associação de Medicina Intensiva Brasileira*, the *Associação Brasileira de Medicina de Emergência*, and the *Sociedade Brasileira de Medicina Hospitalar* formed a group of 17 physicians to formulate questions relevant to the topic and discuss the possibility of consensus for each of them. All questions were prepared using a five-point Likert scale. Consensus was defined a priori as at least 80% of the responses between one and two or between four and five. The consideration of the issues involved two rounds of voting and debate among all participants. The 27 questions prepared make up the present document and are divided into 4 major assessment areas: left ventricular function, right ventricular function, diagnosis of shock, and hemodynamics. At the end of the process, there were 17 positive (agreement) and 3 negative (disagreement) consensuses; another 7 questions remained without consensus. Although areas of uncertainty persist, this document brings together consensus opinions on several issues related to echocardiography in critically ill patients and may enhance its development in the national scenario.

## INTRODUCTION

The echocardiography of critically ill patients has become an essential part of the care provided in the most diverse contexts, from the prehospital environment to the intensive care unit (ICU).^(1)^ Its use as a diagnostic or monitoring tool has gained acceptance in different settings and is endorsed by several international medical entities.^(2-4)^

Echocardiographic evaluation is the second most frequent application of ultrasound in Brazilian intensive care units.^(5)^ Zieleskiewicz et al.^(6)^ reported even higher prevalence rates in a similar European study. The wide use of echocardiography by nonechocardiographers is related to several relevant aspects, both from the organizational and educational point of view and in terms of safety and quality of care. Therefore, it is imperative that medical associations representing the specialties that use echocardiography for the care of critically ill patients analyze the available evidence so that recommendations can be generated that take into account the particularities of the national scenario.

The choice of elaborating a document in consensus format is due to several factors, such as the wide use of echocardiography by nonechocardiographers in the most diverse settings in which critically ill patients are cared for; the wide variation in regional practice in several aspects;^(5)^ the demand by the different medical entities involved that there be guidance on the teaching practices and respective competencies for the use of ultrasound by the nonechocardiographer physician, with a presumed gain in care quality; the scarcity of high-quality evidence to guide the process of escalation of recommendations; and the lack of a similar position in the national scenario that represents the Brazilian reality, in terms of health system organization, professional training, and availability of equipment.^(7)^

The objective of this document is to organize and make available expert consensus opinions that may help clarify the role of bedside echocardiography performed by nonechocardiographers responsible for the care and evaluation of critically ill patients. The present text is complementary to the one that primarily addresses the recommended skills for the use of this tool. Despite related and important intentions, the authors understood that a better definition of the scope of this work would bring agility and consistency to the final document.

## METHODS

This is a collaborative initiative between the *Associação de Medicina Intensiva Brasileira* (AMIB), the *Associação Brasileira de Medicina de Emergência* (ABRAMEDE), and the *Sociedade Brasileira de Medicina Hospitalar* (SOBRAMH).

The committee was initially composed of representatives of each of the entities and later was structured through the appointment of representatives of each of the entities involved. Each member nominated had to be a medical professional and have recognized experience in the use of ultrasound for cardiovascular evaluation in their daily clinical practice. The publication of clinical research in this area and the practice of teaching ultrasound to medical professionals or students in training were recommended criteria, although not mandatory. The final group was formed in February 2019, consisting of 17 consultants representing the collaborative specialties and from different regions of Brazil. All group members completed a declaration of potential conflicts of interest.

The questions were selected using the Delphi method.^(8)^ Two of the authors prepared a set of questions that were submitted electronically to three cycles of judgment by the group. A facilitator assessed the agreement between the individuals and provided individual feedback to each of the consultants about their responses and any questions they might have. Between the second and third consultation cycles, there were no changes in the content of the questions, thus validating them. There were no face-to-face or virtual meetings for this purpose. The 27 validated questions were divided into four broad areas according to the similarity between the specific topics: assessment of left ventricular (LV) function, assessment of right ventricular (RV) function, diagnostic evaluation of shocks, and hemodynamic evaluation. To follow up on the consensus process, the modified Delphi method was used, as described below.

To compile a theoretical basis for obtaining answers to the chosen questions, a systematic review was independently performed in the PubMed database for each of the four major areas by two authors. The structured search strategy for one of the major areas can be found in full in Appendix 1. Each author gathered original studies on the topics of interest, in Portuguese and English, from the date of inception of the database to August 15, 2019. The search was re-run on September 1, 2020. Review articles, letters, editorials, and studies in experimental models were rejected. The set of retrieved articles was rid of duplicates. The set of references that constituted the final product of each search was made available via e-mail to the committee members. Additional consideration of the references of the included articles or of individual searches by each consultant was allowed whenever considered necessary by each member of the committee.

The questions were made available to the committee through an electronic form (Google Forms). All questions were answered on a five-point Likert scale: strongly disagree (1), disagree (2), neutral (3), agree (4), and strongly agree (5). For each question analyzed, the committee members took into account aspects such as consistency of the available evidence, analysis of risks, and benefits, associated costs, learning curve and other barriers to the implementation of bedside echocardiography in each specific scenario. *A priori* consensus was defined as at least 80% of responses being 1 - 2 or 4 - 5.

The facilitator assessed the coherence of the responses obtained from each member. In case of the identification of inconsistency between the responses that suggested an error in the understanding of the statement or a mistake in filling out the questionnaire, he sent individual responses by e-mail as a form of conference. The issues that did not generate consensus in the first round of submissions were forwarded to the members of the advisory committee for a second round, performed 4 weeks after the first round. At the end of each round, all participants received a complete summary of the group voting results for each question evaluated, as well as their own responses. The individual responses of each member were kept confidential from the other members of the committee at all stages of the process.

The issues that still had no consensus after this stage were subjected to online voting in two virtual meetings held in October and November 2020, which brought together all the members of the committee. In this stage, the participants had the opportunity to discuss the particularities of each of the questions and argue for their position. The duties of the facilitator in the first stage consisted of clarifying any doubts the participants had and allowing all participants who wished to do so to have the opportunity to express their views, without the need to reach a consensus on any questions, and to compile the results of the votes obtained on each of the questions.

In the virtual meetings, the questions lacking consensus after the first two stages were presented to the participants in a grouped manner in two groups: first, questions close to consensus, meaning those that had more than 60% of the answers concentrated in 1 - 2 or 4 - 5); and second, the questions far from consensus, which had responses that were less than 60% 1 - 2 or 4 - 5. The votes were also obtained anonymously through the online platform Mentimeter (www.mentimeter.com). After the online voting results, questions that had not yet reached consensus were put to a new vote only once if the absolute majority of participants agreed.

## RESULTS

All participants answered the questions relevant to each stage, including at the virtual meeting, with the exception of the facilitator. Thus, the other 16 responses were summed for all questions. In the first round, consensus was reached on 14 of the 27 questions: one of seven in the LV systolic function domain, three of the six in the RV systolic function domain, all six in the shock assessment domain, and four out of eight in the hemodynamic evaluation domain. In the second round, two other questions reached consensus, leaving 11 questions for virtual-meeting discussion among the participants. At the end of all steps, there were 17 positive (agreement) and three negative (disagreement) consensuses; another seven questions never reached consensus among the participants, overrepresented in the domains LV function and hemodynamic evaluation (three questions each) (Table 1).

**Table 1:** Questions addressed and their degrees of agreement on the five-point Likert scale

	Consensus stage		Strongly disagree		Disagree		Neutral	Agree		Strongly agree	
**Assessment of LV systolic function**											
1. Qualitative assessment of global LV function is the preferred way of assessing critically ill patients by nonspecialist physicians	1		0		0		0	1		15	
				0%			0%		100%		
2. Quantitative assessment of LV function in critically ill patients may be performed by nonspecialist physicians in selected situations	2		1		2		0	4		9	
				18,75%			0%		81,25%		
3. The Simpson method is the method of choice for the quantitative assessment of LV function in critically ill patients by nonspecialist physicians.	3		11		2		0	1		2	
				81,25%			0%		18,75%		
4. dP/dT should be used by nonspecialist physicians for semiquantitative evaluation of LV systolic function	3		11		3		2	0		0	
				87,5%			12,5%		0%		
5. The Teichholz method is the method of choice for the quantitative assessment of LV function in critically ill patients by nonspecialist physicians	No		7		2		2	2		3	
				56,25%			12,5%		31,25%		
6. MAPSE should be used by nonspecialist physicians for semiquantitative evaluation of LV systolic function	No		1		1		3	5		6	
				12,5%			18,75%		68,75%		
7. The S’ wave should be used by nonspecialist physicians for semiquantitative evaluation of LV systolic function	No		3		3		3	4		3	
				37,5%			18,75%		43,75%		
**Assessment of RV systolic function**											
8. An assessment of RV function should be routinely performed in situations of severe hypoxemia and ARDS	1		0		0		1	2		13	
				0%			6,25%		93,75%		
9. An evaluation of RV function should be routinely performed in cases of PTE	1		0		0		0	1		15	
				0%			0%		100%		
10. The assessment of RV function by nonspecialists should be performed using the parameters of global systolic function (RV/LV dimensions, interventricular septal dynamics)	1		0		0		0	2		14	
				0%			0%		100%		
11. The assessment of RV function by nonspecialists should be performed by measuring FAC	3		10		3		2	0		1	
				81,25%			12,5%		18,75%		
12. The assessment of RV function by nonspecialists should be performed by measuring the parameters of longitudinal function (TAPSE, S’ wave)	2		1		0		1	5		9	
				6,25%			6,25%		87,5%		
13. The assessment of RV function by nonspecialists can be performed by measuring right chamber pressures in selected situations	No		3		4		2	3		4	
				43,75%			12,5%		43,75%		
**Diagnostic evaluation of shocks**											
14. Bedside echocardiography should be routinely used in the initial evaluation of shocks.	1		0		0		0	1		15	
				0%			0%		100%		
15. Bedside echocardiography should be routinely used in the follow-up of shocks and in the reassessment after institution of therapies.	1		0		0		0	1		15	
				0%			0%		100%		
16. Bedside echocardiography contributes to the recognition of severe hypovolemia as a cause of shock	1		0		0		0	1		15	
				0%			0%		100%		
17. Bedside echocardiography contributes to the recognition of *cor pulmonale* as the cause of shock	1		0		0		0	1		15	
				0%			0%		100%		
18. Bedside echocardiography contributes to the recognition of cardiac tamponade as a cause of shock	1		0		0		0	0		16	
				0%			0%		100%		
19. Bedside echocardiography contributes to the recognition of severe LV dysfunction as a cause of shock	1		0		0		0	0		16	
				0%			0%		100%		
**Hemodynamic evaluation**											
20. The estimation of central venous pressure through echocardiography by a nonspecialist physician is recommended as part of the hemodynamic evaluation of critically ill patients	3		1		0		2	3		10	
				6,25%			12,5%		81,25%		
21. The estimation of left atrial pressure by means of echocardiography by a nonspecialist physician is recommended as part of the hemodynamic evaluation of critically ill patients.	No		3		3		1	3		6	
				37,5%			6,25%		56,25%		
22. Estimation of extravascular pulmonary water by means of chest ultrasound by a nonspecialist physician should be part of the hemodynamic evaluation of critically ill patients.	1		2		0		0	2		12	
				12,5%			0%		87,5%		
23. B-lines on lung ultrasound can be used as a safety measure for fluid delivery	1		0		1		2	4		9	
				6,25%			12,5%		81,25%		
24. Inferior vena cava variability should be used as a tool to assess fluid responsiveness	No		2		1		2	3		8	
				18,75%			12,5%		68,75%		
25. Functional hemodynamic tests (minibolus and final respiratory occlusion test) should be used as a tool for assessing fluid responsiveness	No		4		2		0	8		2	
				37,5%			0%		62,5%		
26. The passive leg elevation maneuver should be used as a tool to assess fluid responsiveness	1		0		1		0	6		9	
				6,25%			0%		93,75%		
27. The estimation of CO through the measurement of the velocity-time integral should be used as a tool for hemodynamic evaluation	1		0		0		0	5		11	
				0%			0%		100%		

LV - left ventricle; dP/dT - rate of change in pressure per time interval; MAPSE - Mitral annulus plane systolic excursion ; RV - right ventricle; ARDS - acute respiratory distress syndrome; PTE - pulmonary thromboembolism; FAC - fractional area change ; TAPSE - measurement of the systolic excursion of the tricuspid annulus plane; CO - cardiac output.

To enable the reader to become familiar with the technique for obtaining images by means of echocardiography to better understand the aspects discussed here, we will briefly describe the main echocardiographic windows used in the bedside examination.

### Long (or longitudinal) parasternal window

**Figure 1:**
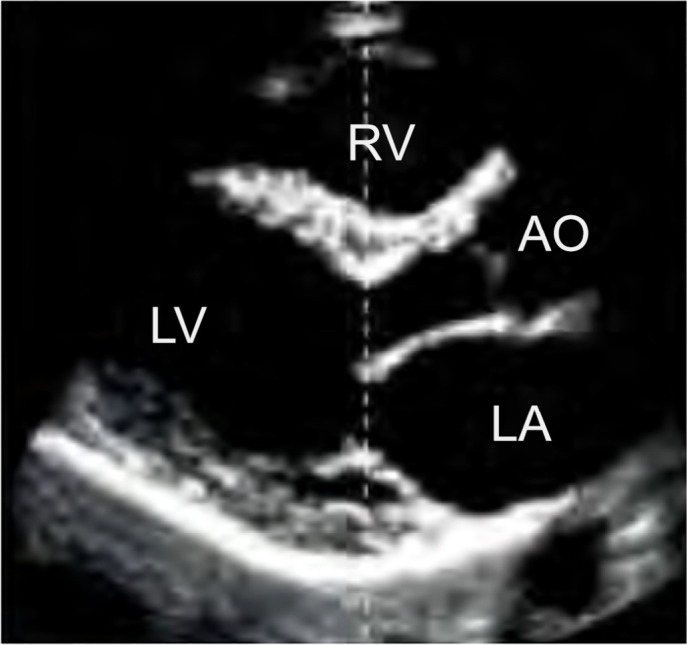
Parasternal longitudinal window.

With the transducer positioned near the left sternal border, in the second or third intercostal space, and with the marker directed to the patient’s right shoulder, the main structures visualized in this window can be identified: RV, interventricular septum, LV, inferolateral wall, mitral and aortic valves, and left atrium (Figure 1). Through this view, it is possible to obtain important information, such as the relationship between RV and LV and LV systolic function.

RV - right ventricle; LV - left ventricle; AO - aorta; LA - left atrium.

Source: adapted from Barros DS, Bravim B. *Ecografia em terapia intensiva e na medicina de urgência*. São Paulo: Atheneu; 2018.

### Short (or transverse) parasternal window

**Figure 2:**
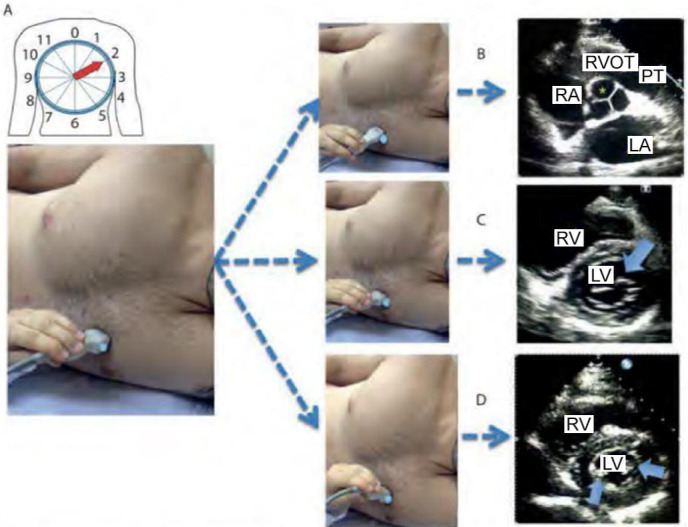
Several observation planes in the transverse parasternal window. (A) Patient in the left lateral decubitus position. Transducer in the third left intercostal space, with the index pointed to the left shoulder (2 hours). (B) Transducer with tip tilted upward to visualize the section at the level of the aortic valve (see asterisk). (C) Less inclined transducer, obtaining a section at the level of the mitral valve (see arrow). (D) Transducer with tip inclined downward, visualizing the section at the level of the papillary muscles (see arrows).

Keeping the transducer positioned in the same location where the longitudinal view was obtained, the examiner performs a rotation of approximately 90°, now directing the marker to the patient’s left shoulder (Figure 2). Depending on the height above the LV at which the slice is obtained, different structures may be evaluated. At the level of the papillary muscles, the RV and LV are identified; with a slight cranial inclination, the mitral valve is added. In an even more cranial plane, at the level of the aortic valve, we can identify the left atrium, right atrium, tricuspid valve, RV, pulmonary valve, and, eventually, the pulmonary artery and its main branches. The short parasternal window has among its main applications the global and segmental assessment of LV systolic function, as well as the dynamics between RV and LV.

RVOT - right ventricular outflow tract; PT - pulmonary artery trunk; LA - left atrium; RA - right atrium; RV - right ventricle; LV - left ventricle.

Source: Barros DS, Bravim B. *Ecografia em terapia intensiva e na medicina de urgência*. São Paulo: Atheneu; 2018.

### Apical window

**Figure 3:**
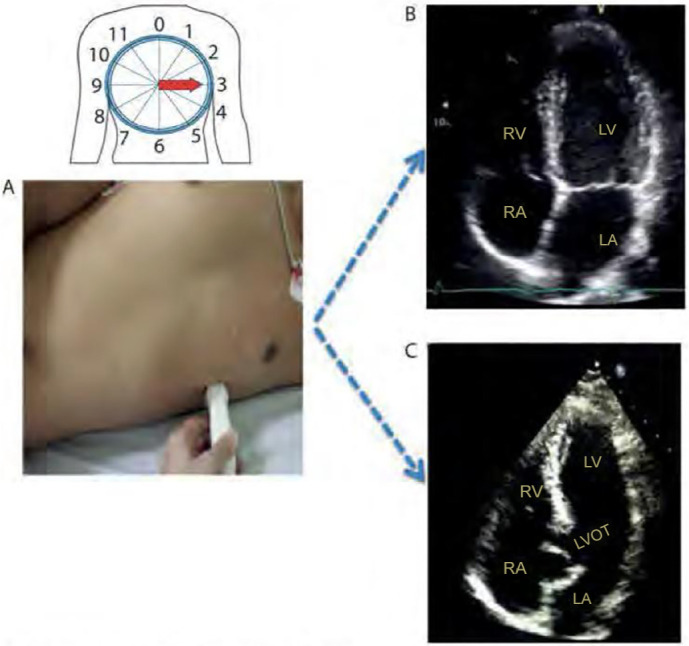
Fourand five-chamber apical windows. (A) Patient in the left lateral semidecubitus position (slightly inclined toward the back). Transducer in the fifth left intercostal space, between the midclavicular line and the anterior axillary line, with the index pointed to the left arm (3 o’clock). (B) Four-chamber apical window. (C) Apical five-chamber window: obtained from the apical four-chamber window, with the tip of the transducer tilted slightly upward, maintaining contact with the patient’s skin, in which the aortic valve and the left ventricular outflow tract can be seen.

By placing the transducer close to the cardiac apex, or approximately in the fifth or sixth intercostal spaces, with the marker pointing to the patient’s left arm, the apical view is obtained. The four chambers of the heart are identified: the two atria and the two ventricles (Figure 3). The apical window is of fundamental importance for many of the quantitative measurements obtained in bedside echocardiography through the application of the Doppler effect because it provides a better alignment of the transducer in relation to the systolic and diastolic flows between the cardiac chambers. A light cranial scan of the transducer will allow the operator to visualize the LV outflow tract (known as the “fifth chamber”, now characterizing the apical five-chamber view). The main applications of the five-chamber apical view are the evaluation of the morphology and functionality of the aortic valve and the acquisition of the velocity-time integral (VTI), used in the estimation of cardiac output (CO) obtained by echocardiography.

RV - right ventricle; LV - left ventricle; LA - left atrium; RA - right atrium; LVOT - left ventricular outflow tract.

Source: Barros DS, Bravim B. *Ecografia em terapia intensiva e na medicina de urgência*. São Paulo: Atheneu; 2018.

### Subcostal window

**Figure 4:**
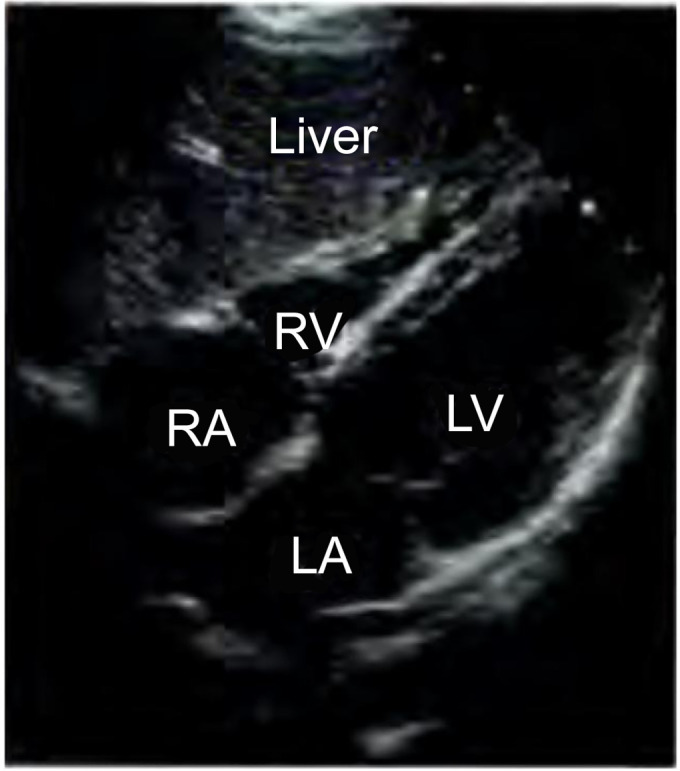
Four-chamber subcostal window, where the liver can also be visualized.

With the transducer positioned approximately 1 to 2 cm below the xiphoid process and the index finger still directed toward the patient’s left arm, a four-chamber subcostal view can be obtained, in which the two atria and two ventricles are also identified, although in a different orientation than that obtained in the apical sections (Figure 4). The evaluation of structures in this view is limited in some aspects, mainly due to their orientation in relation to the transducer. However, in patients undergoing mechanical ventilation (MV) or with pulmonary emphysema, for example, it may be the option that gives the best image quality. One of its characteristics is that it allows the investigation of pericardial effusion, precisely because of its approach to the dependent side of the heart.

RV - right ventricle; LV - left ventricle; LA - left atrium; RA - right atrium.

Source: Barros DS, Bravim B. *Ecografia em terapia intensiva e na medicina de urgência*. São Paulo: Atheneu; 2018.

**Figure 5:**
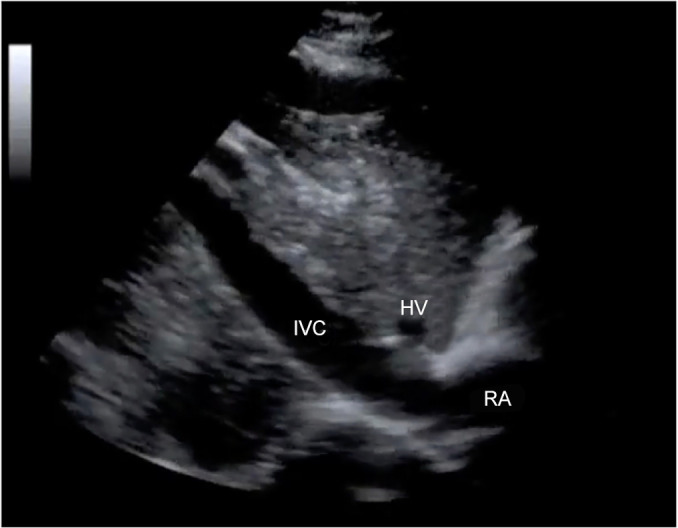
Subcostal window of the inferior vena cava.

A light caudal sweep can identify the inferior vena cava (IVC) in cross-sectional view. On the other hand, starting from the subcostal view with the right atrium at the center of the image, a rotation of the transducer positioning the index finger toward the sternal notch, the IVC can be visualized in a longitudinal position (Figure 5). These views allow the evaluation of its diameter as well as its degree of variation induced by ventilation.

IVC - inferior vena cava; HV - hepatic vein; RA - right atrium

Source: Authors’ personal collection.

#### Domain 1 - Assessment of LV systolic function

**1. Qualitative assessment of global LV function is the preferred way of assessing critically ill patients by nonspecialist physicians - 100% agreement.**

**2. The quantitative assessment of LV function in critically ill patients can be performed by a nonspecialist physician in selected situations - 81.25% agreement.**

The qualitative assessment of LV global function is often used in the evaluation of critically ill patients. Several authors called eye-balling the act of determining ventricular function through visual inspection, without the use of any quantitative method. Eye-balling can be performed more quickly than quantitative reference methods^(9)^ while eliminating the delineation of the endocardial border, which can be laborious and time-consuming, even in patients with a favorable echocardiographic window.

Most published curricula for training in the ultrasonography of critically ill patients recommend the qualitative evaluation of LV function (or even binary evaluation: with or without dysfunction) as the method of choice.^(10)^ Melamed et al. identified a good correlation between the categorization into ejection fraction levels of intensivists with brief immersion training using portable equipment and that of echocardiographers using conventional equipment.^(11)^ The evaluation performed using this approach tends to be more accurate than quantitative assessment.^(12)^

The participants unanimously agreed that the preferred method for assessing LV systolic function should be qualitative, but 81.25% agreed that nonspecialist physicians can use quantitative assessment in selected situations. Kanji et al.,^(10)^ in a systematic review of 15 studies that evaluated ultrasound curricula for critically ill patients, reported that the mean correlation found between nonspecialists and echocardiographers for the qualitative assessment of LV systolic function was 0.67.

**3. The Simpson method is the method of choice for the quantitative assessment of LV function in critically ill patients by nonspecialist physicians - 81.25% disagreement.**

**4. The rate of change of pressure per time interval (dP/dT) should be used by a nonspecialist physician for semiquantitative evaluation of LV systolic function - 87.5% disagreement.**

**5. The Teichholz method is the method of choice for the quantitative assessment of LV function in critically ill patients by nonspecialist physicians - without consensus.**

**6. Mitral annular plane systolic excursion (MAPSE) should be used by nonspecialist physicians for semiquantitative evaluation of LV systolic function - without consensus.**

**7. The S’ wave should be used by nonspecialist physicians for semiquantitative evaluation of LV systolic function - without consensus.**

The evidence regarding the evaluation of the LV in critically ill patients is quite limited, as most of the available studies included patients with structural heart disease, not necessarily in the presence of acute disease.

Bergenzaun et al.^(13)^ evaluated several parameters for the evaluation of LV systolic function in a population of mechanically ventilated critically ill patients in shock. All the parameters studied were feasible in this population, although the uniplanar Simpson method was not obtainable in 7% of the individuals (and it showed an intraobserver variability of 10.6%). The qualitative estimates by eye-balling and MAPSE were obtained in 100% of the patients, and the eye-balling method correlated well with Simpson’s method throughout the study period.

The biplanar Simpson method is widely considered the standard for quantitative assessment of LV ejection fraction.^(14,15)^ Although it may provide useful information for the proper assessment of LV function, it is a time-consuming method, requires acquisition of echocardiographic images that are precise enough to delineate the endocardial border, presents significant intraand interobserver variability in critically ill patients,^(16)^ and demands a near-specialist level of expertise from the examiner. The uniplanar method can be considered an alternative with good correlation with the biplanar method.^(17)^ and greater agility in obtaining them.

The Teichholz formula, although previously widely used to convert diameters into systolic and diastolic volumes (and therefore the ejection fraction), also requires good image resolution and proper alignment of the LV walls for its measurement, and it tends to underestimate the repercussion of regional impairment of ventricular function, especially in patients with structural heart disease.

The use of any of the techniques should take into account the inherent limitations of the ejection fraction itself as a measure of systolic function in critically ill patients.^(18)^ Acute changes in blood volume or in preand afterload, for example, can significantly alter ejection fraction without necessarily implying an effective change in systolic function. For the above reasons, the committee did not reach consensus on issues related to the measurement of ejection fraction.

**Figure 6:**
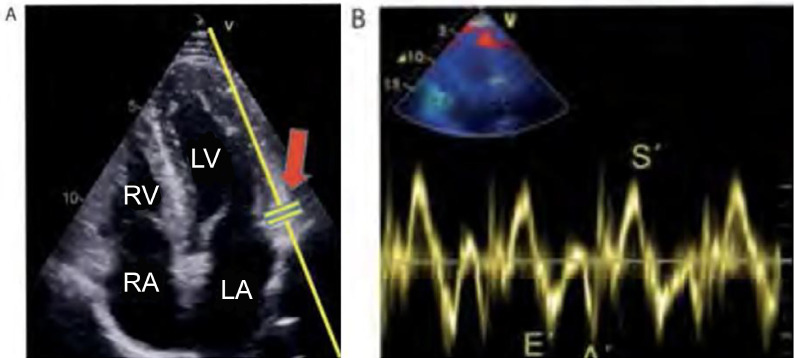
Measurement of tissue Doppler S’ wave. (A) Positioning of the tissue Doppler cursor on the lateral wall of the mitral annulus (arrow) in the apical four-chamber view. (B) Tissue Doppler curve in a patient with normal systolic function, in which we can visualize the systolic wave and the E’ and A’ diastolic waves. Peak velocity of the S’ wave with normal amplitude (S’ wave > 9cm/s).

Regarding the other evaluation parameters of LV function, neither the use of MAPSE nor the measurement of the S’ wave by means of tissue Doppler (Figure 6) was met with consensus. Although they may detect more subtle changes in ventricular function,^(19)^ they are mostly tested in studies of noncritical patients^(20,21)^ and demand an adequate alignment of the image to avoid underestimation. The MAPSE measurement may constitute a viable alternative in patients with unfavorable acoustic windows.^(22)^ In patients in shock, the reduction in MAPSE was correlated with mortality at 28 days.^(23)^ Despite the favorable aspects considered above, both the acquisition of the MAPSE and the S’ wave require a certain degree of expertise on the part of the operator, so that there are no errors in the acquisition of the image and thus in its interpretation and in the subsequent decision-making. We believe that the lack of consensus observed on these topics is related to the fact that they are inherently quantitative measures, in contrast to those qualitative parameters and subjective global assessments that characterize the essence of bedside echocardiography by the nonechocardiographer physician.

LV - left ventricle; LA - left atrium; RA - right atrium; RV - right ventricle.

Source: adapted from Barros DS, Bravim B. *Ecografia em terapia intensiva e na medicina de urgência*. São Paulo: Atheneu; 2018.

The evaluation by means of the dP/dT, although validated for a long time in the population of noncritical individuals,^(24,25)^ requires the identification of mitral regurgitation flow and lacks evidence in acutely ill patients, in addition to demanding from the operator all the above-described requirements of adequate alignment and image resolution. Thus, the committee members took a position contrary to the routine employment of this parameter by the nonspecialist physician (87.5% disagreement).

#### Domain 2 - Assessment of RV systolic function

**8. An assessment of RV function should be routinely performed in situations of severe hypoxemia and acute respiratory distress syndrome (ARDS) - 93.75% agreement.**

**9. An assessment of RV function should be routinely performed in cases of pulmonary thromboembolism (PTE) - 100% agreement.**

Since Jardin et al.,^(26)^ the evaluation of RV function has received greater attention due to its fundamental role in different scenarios commonly encountered in the care of critically ill patients. The first decade of the 2000s marked an exponential increase in publications involving echocardiographic evaluation of the RV in critically ill patients, as the greater availability of portable machines in intensive care units raised interest in its role.^(27)^

Right ventricular failure should be considered a heterogeneous syndrome, not a specific condition. Although the generic prevalence of RV failure in critically ill patients has not been established, some contexts seem to be more frequently present: Patients who are hypoxemic of any nature, patients with myocardial dysfunction associated with sepsis, and patients in shock are at increased risk of RV failure.^(28)^

Mechanical ventilation with positive pressure, by itself, is associated with impairment of RV function, and among the effects on the RV, the increase in afterload and reduction of preload stand out.^(29)^ The magnitude of the effects of invasive MV (IMV) on the RV is related to chest compliance, tidal volume, and right ventricular positive end-expiratory pressure (PEEP) applied, among other factors. Fougères et al.^(30)^ demonstrated that the increase in PEEP from 5cmH_2_O to the mean value of 13cmH_2_O (or the highest PEEP, reaching 30cmH_2_O plateau pressure) was accompanied by an increase in RV end-diastolic diameter and vascular resistance lung function and a decrease in CO.

Acute respiratory distress syndrome is one of the clinical situations that most commonly poses challenges to RV function due to the acute increase in afterload. These patients present not only alveolar involvement and hypoxemia but also structural changes in the pulmonary circulation that progress with inflammation, vasoconstriction, edema and microthrombi, culminating in an increase in pulmonary artery pressure.^(31)^ The prevalence of acute *cor pulmonale* has been reported as up to 25% in patients with ARDS,^(32,33)^ although it was 60% when the MV protocol used higher inspiratory volumes and pressures than the current practice.^(34)^

Hypercapnia, elevation of driving pressure above 18 mmHg and plateau pressure are associated with the development of RV failure.^(35)^ The fact that the ventilatory strategy seems to interfere with RV performance led the authors to put forth strategies designated “RV protection”, limiting the plateau pressure, driving pressure, and partial pressure of carbon dioxide (PaCO_2_), in addition to limiting the plateau pressure, driving pressure, and partial pressure of carbon dioxide (PaCO_2_), resorting to prone ventilation when these goals are not achieved. Prone ventilation seems to be associated with relief of pressures on the right side of the heart, as demonstrated by Vieillard-Baron et al.^(36)^ in a study that included 42 individuals with severe ARDS and that found that both the RV dimensions and septal dyskinesia are attenuated after an 18-hour session in the prone position. Accordingly, Joswiak et al.^(37)^ reported a reduction in the RV:LV ratio, a reduction in the eccentricity index, and an increase in CO.

Dynamic parameters should be used to assess fluid responsiveness with caution in patients with RV dysfunction, as the chance of false-positives increases in this situation, and volume expansion can result in hemodynamic deterioration through the mechanisms of ventricular interdependence. The evaluation of echocardiographic parameters of RV function before and after volume delivery can be used to rule out the development of acute RV failure.^(29,38)^

Patients with chronic obstructive pulmonary disease (COPD) are at increased risk of developing RV overload, especially when the COPD is exacerbated and they are subjected to MV. Up to 80% of COPD patients will show signs of overload, whether of a chronic or a acute nature.^(39)^ Up to one-third of patients with pulmonary embolism will have signs of RV distress.^(40)^ A similar prevalence can be found in inferior infarction. ^(41)^ Regardless of the etiology, the identification of RV distress in critically ill patients has prognostic relevance in settings such as ARDS,^(33)^ PTE^(40)^ and myocardial infarction,^(42,43)^ resulting in higher mortality.

There was a consensus that the RV should be evaluated by a nonspecialist physician in ARDS and PTE situations (93.75% and 100%, respectively). However, the evaluation of RV functionality may be important in several scenarios often found in ICUs and emergency rooms. The present document is not intended to exhaust the diagnostic possibilities of bedside echocardiography; the narrowing of the scope of the questions favored the understanding of the committee members and allowed for a consistent position on several questions in this and other evaluated domains; and specific situations, such as RV infarction, pulmonary hypertension, and congenital heart disease, although also frequent, may require specialized evaluation of RV function, being at the border of the possibilities of bedside echocardiography by a nonechocardiographer physician.

Not surprisingly, in a considerable number of critically ill patients, it will not be possible to assess RV function using the transthoracic approach: Huang et al.^(27)^ reported failure rates of up to 27% of individuals to obtain adequate measurements.

The functional approach to the RV is challenging, both because of its pyramidal shape and because of its retrosternal anatomical location and its condition that depends on the preload of most parameters used for its evaluation.^(44)^ Furthermore, RV function may be directly influenced by ventilatory strategies, volume expansion, or vasoactive drugs, making its evaluation essential for the best treatment of critically ill patients.

Ideally, right heart chamber pressures are measured invasively, either through conventional right catheterization in the hemodynamics laboratory or by insertion of a pulmonary artery catheter, even allowing continuous monitoring of pulmonary artery pressure. Echocardiography is a useful (and even complementary) alternative for the evaluation of the right chambers, both because of its noninvasive nature and because it allows the integration of morphological aspects, chamber dimensions, and functional parameters.

Huang et al.^(27)^ recently published an extensive systematic review addressing all the parameters of RV function described in critically ill patients in the ICU, operating room, or emergency department, including, for the most part, patients with PTE, ARDS, postoperative cardiac surgery, and myocardial dysfunction combined with sepsis. Studies of prognosis (28%) and associations between variables (27%) prevailed. Most studies (69%) used a combination of parameters to assess RV function. Although the use of a single parameter results in greater simplicity, each parameter has specific advantages and limitations and may not be ideal for the clinical situation or patient in question.

The parameters of RV function can be classified as global function, longitudinal function, and right chamber pressure.

**10. The assessment of RV function by nonspecialists should be performed using the parameters of global systolic function (RV/LV dimensions, interventricular septum dynamics) - 100% agreement.**

### Global function parameters

#### Measurement of RV and RV dimensions/EV

Although reference values for RV dimensions are not adequately validated for patients under VM, their comparison with the left side can serve as a reference.

The planimetry of the endocardial edge of both ventricles in the apical four-chamber view to measure their respective areas can be used for this purpose.^(45)^ The relationship between the RV and LV areas is commonly used in the definition of *cor pulmonale* with anomalous septal movement.^(27)^ Under physiological conditions, the RV diastolic area will be up to 60% of the LV diastolic area (RV/EV up to 0.6). When the RV area exceeds 60% of the LV, there will be RV dilation, which is considered severe if the RV/LV ratio is greater than 1 (RV greater than LV). Vieillard-Baron et al.^(46)^ found a mortality rate of 25% in patients with ARDS and an RV area ratio/EV greater than 1.

Additionally, using the apical four-chamber view, it is possible to measure the distance between the interventricular septum and the lateral insertions of the tricuspid and mitral rings, yielding the RV and LV diameters, respectively. The same parameters for RV/EVs used for the area may be used with their diameters. One-dimensional measurements, however, may have limited accuracy under conditions of increased RV preand afterload.^(47)^

In obtaining these measurements, special care should be taken to measure the largest possible RV dimensions, as window angle distortions are frequent causes of underestimation. These measurements should be performed at the end of ventricular diastole, with the atrioventricular valves at their maximum openness.

### Evaluation of the interventricular septum dynamics

The interventricular septum is part of the anatomical structure of the LV and should maintain, together with the other LV walls, a symmetrical conformation, with synchronous contractility in the transverse axis. This, however, depends on the maintenance of physiological pressure relationships.

In situations of an increase in pressure on the right side of the heart, the interventricular septum may be pushed back toward the LV, becoming straightened in some or all of the cardiac cycle. Dyssynchronous contraction of the septum relative to the remainder of the LV is termed paradoxical movement and should be considered a specific sign of increased RV afterload. Up to 22% of patients with ARDS exhibit paradoxical septal movement within the first 3 days of ARDS.^(35)^

**11. The assessment of RV function by a nonspecialist should be performed by measuring the fractional area change (FAC) - 81.25% disagreement.**

### Fractional area change

Based on the planimetry of the RV endocardial border at end-systole and end-diastole, its fractional change can be calculated as (diastolic area - systolic area)/diastolic area. Fractional area change values < 35% indicate RV dysfunction. Fractional area change is associated with RV ejection fraction and is even used in some studies as a parameter of comparison for other indices.^(48)^ The reduced rate also has prognostic importance: independent of other factors, it was associated with all-cause mortality in patients after myocardial infarction.^(49)^ For proper measurement, it is necessary to carefully and manually delimit the endocardial border, starting from the lateral tricuspid annulus, following the RV free wall to the medial tricuspid annulus, which can be technically challenging in situations of inadequate positioning (when the decubitus position is exclusively dorsal), IMV, and use of dedicated bedside equipment, which is not always sufficient to perform advanced echocardiographic measurements. Furthermore, it should be noted that while the measurement incorporates septal contractility (and is therefore influenced by the LV), the contribution of the RV outflow tract will not be taken into account. For these reasons, the committee members opposed the routine use of this parameter.

**12. The assessment of RV function by nonspecialists should be performed by measuring the parameters of longitudinal function (tricuspid annular plane systolic excursion [TAPSE], S’ wave) - 87.5% agreement.**

### Longitudinal function parameters

#### Measurement of the systolic excursion of the tricuspid annulus plane

**Figure 7:**
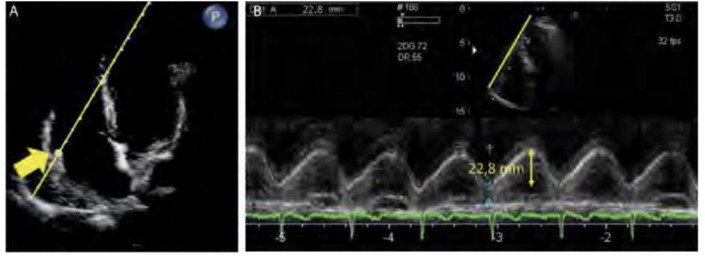
Measurement of the systolic excursion of the tricuspid annulus plane. (A) Positioning of the M-mode cursor at the level of the lateral base of the tricuspid annulus (arrow) in the four-chamber apical window. (B) M-mode waveform depicting the movement of the lateral base of the tricuspid ring during the cardiac cycle. The ascending phase of the tracing corresponds to systole. The systolic excursion of the tricuspid annulus plane is measured as the height of the wave. In this patient, the systolic excursion of the tricuspid annulus plane was 22.8mm (normal value > 17mm).

Source: Barros DS, Bravim B. *Ecografia em terapia intensiva e na medicina de urgência*. São Paulo: Atheneu; 2018.

The arrangement of the myocardial fibers in the RV follows a predominantly longitudinal orientation, as opposed to the LV, which is transversal. Thus, the main mechanism of RV contraction occurs in the long axis, from the base toward the apex. The maximum displacement of the tricuspid plane toward the RV apex can be measured using the M-mode (Figure 7).

The TAPSE value is related to the RV ejection fraction measured by myocardial scintigraphy.^(50)^ When below 17 mm, it suggests RV dysfunction and has prognostic impact in different scenarios,^(51-53)^ being an isolated predictor of mortality in a recent study of patients with ARDS.^(54)^ TAPSE does not provide information on regional contractility and may be inaccurate in cases of segmental dysfunction.

Tricuspid annular plane systolic excursion is the parameter most frequently studied in critically ill patients, possibly due to the simplicity of its measurement. It is, however, subject to distortions, especially in relation to the measurement axis and movement artifacts of the heart and the patient himself. It is essential to pay attention to the correct alignment of the ultrasound beam with the axis of longitudinal contraction of the RV to avoid underestimation. In this way, good intraand interoperator reproducibility can be obtained.^(27)^

#### Measurement of tricuspid S’ wave

**Figure 8:**
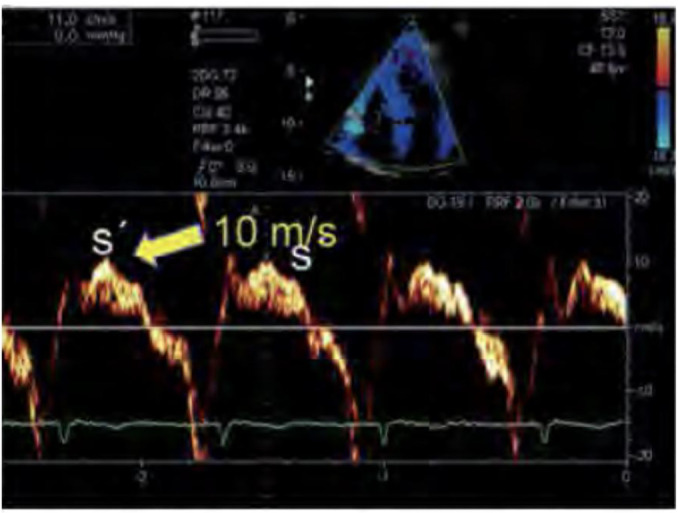
Tissue Doppler imaging of the peak velocity of tricuspid annulus displacement during right ventricular systole (tissue S’ wave).

Source: adapted from Barros DS, Bravim B. *Ecografia em terapia intensiva e na medicina de urgência*. São Paulo: Atheneu; 2018.

In addition to TAPSE, the application of tissue Doppler imaging on the tricuspid annulus, together with its insertion into the RV free wall, allows the measurement of the maximum velocity of myocardial displacement toward the apex, representing a parameter of systolic function (Figure 8). An S’ wave value below 10 cm/s is considered indicative of RV dysfunction.

As with TAPSE, attention should be paid to artifacts of movement and angulation of the longitudinal axis. The S’ wave value depends less on the quality of the image obtained in B-mode, allowing measurements even with limited windows.

Although correlated with pulmonary artery systolic pressure (PASP) measurements obtained using the tricuspid regurgitation jet, this method still lacks validation against invasive measurements using right heart catheterization.^(55)^ In critically ill patients, S’ wave measurement is not as widely used as TAPSE, but it has been associated with prolonged MV,^(56)^ the severity of sepsis, and its prognosis.^(57)^

**13. The assessment of RV function by nonspecialists can be performed by measuring right chamber pressures in selected situations - without consensus.**

### Right chamber pressures

#### Pulmonary artery systolic pressure via the tricuspid regurgitant jet

**Figure 9:**
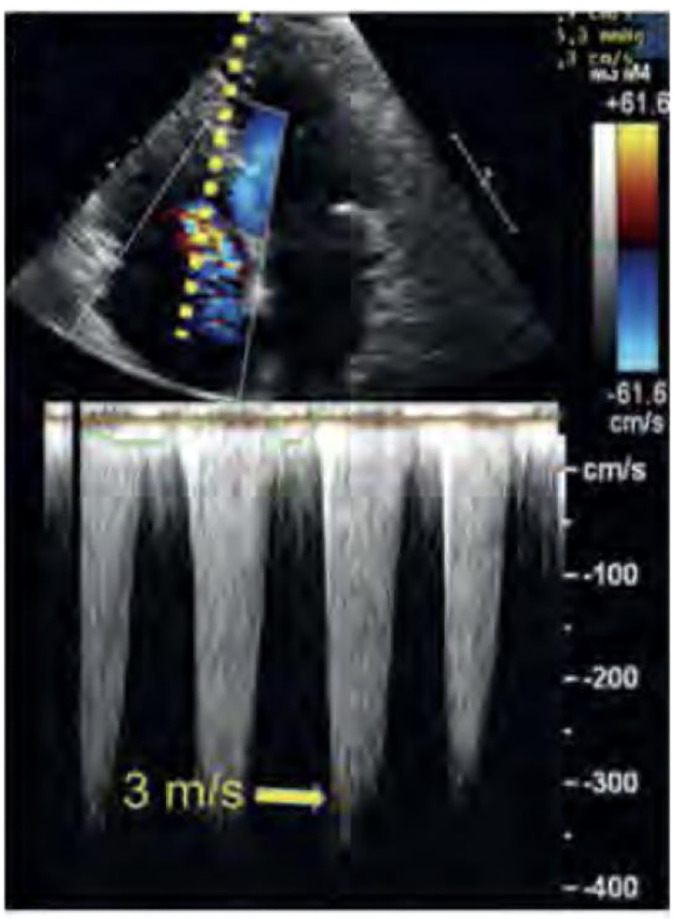
Estimated maximum velocity of tricuspid regurgitation (approximately 3m/sec). First, we must locate the jet with color Doppler imaging. Next, we align the Doppler cursor (dashed line) with the jet and select the continuous Doppler function. Then, in the speed record, a continuous curve appears.

Source: adapted from Barros DS, Bravim B. *Ecografia em terapia intensiva e na medicina de urgência*. São Paulo: Atheneu; 2018.

Unlike the mitral valve, the tricuspid valve may dilate in its lateral axis in response to downstream pressure elevations, decompressing an RV under pressure overload, although it may result in upstream congestion and reduced LV preload.^(28)^ The evaluation of the tricuspid regurgitant jet provides information about the degree of elevation of the pressures in the pulmonary arterial bed: as a rule, the maximum velocity of tricuspid regurgitation is directly proportional to the pulmonary arterial pressure. A regurgitation velocity of less than 2 m/s is considered normal (Figure 9).^(58)^

With the use of continuous Doppler aligned to the axis of the regurgitant jet, the simplified Bernoulli formula [4(V_max_)^^2^^] allows the calculation of the pressure gradient from the direct measurement of the maximum regurgitant velocity.^(59)^ This gradient should then be added to the right atrial pressure (RAP) to result in the estimation of PASP (see Domain 4, Hemodynamic assessment - estimation of central venous pressure).

The agreement between the PASP measurement using the Bernoulli equation and right catheterization is moderate,^(60)^ since this method assumes that there is a direct transformation of potential energy (pressure gradient) into kinetic energy (peak velocity of the tricuspid regurgitation jet). In situations where this relationship is altered, the pressure estimate may be consequently affected. Eccentric regurgitant jets or patients with a small RA may have an underestimated peak pressure. Furthermore, factors such as marked dilation of the tricuspid annulus (and consequent continuous RV-RV reflux, with potential equalization of pressures), as well as RV systolic dysfunction, imply a risk of underestimation if the measures dependent on the analysis of tricuspid regurgitation flow. Likewise, polycythemia or severe anemia can interfere with blood viscosity and result in underestimation or overestimation, respectively.^(61)^ Considering that the regurgitation velocity factor will be squared, small measurement errors will result in substantially different measurements.

Most of the studies that analyzed the agreement between echocardiographic parameters and invasive measures of PASP were performed in stable patients under spontaneous ventilation. In situations where there is lung hyperinflation (MV or COPD, for example), the accuracy of these parameters is less known. Arcasoy et al.^(62)^ reported significant deviations from this measure in patients with advanced lung disease on the list for lung transplantation. In critically ill patients undergoing IMV and monitoring with a pulmonary artery catheter, Bouhemad et al.^(63)^ reported a significant correlation (r = 0.74) between tricuspid regurgitation and PASP. More recently, Mercado et al.^(64)^ reported a significant correlation (r = 0.87) with PASP and 100% accuracy for the identification of pulmonary hypertension.

The proportion of patients in whom it is feasible to evaluate tricuspid regurgitation is approximately 75% among outpatients^(65)^ and between 60 and 70% among critically ill patients on MV^(63,64)^ due to the presence of obstacles such as an insufficient cardiac window and hyperinflation.^(66)^ The effective absence of tricuspid regurgitation, despite making this approach impossible, does not rule out elevation of pulmonary artery pressure: approximately 20% of patients with PASP above 35mmHg will not have tricuspid regurgitation; among those with PASP above 50, up to 95% will have a detectable regurgitant jet.^(60)^

### Mean pulmonary artery pressure

Mean pulmonary artery pressure (MPAP) is an essential parameter for the calculation of pulmonary vascular resistance, in addition to being representative in the evaluation of scenarios in which pulmonary hypertension is suspected. This pressure can be measured in different ways by means of echocardiography, mainly the evaluation of the pulmonary regurgitant jet, the acquisition of the VTI through planimetry of the tricuspid regurgitant jet, and the measurement of the acceleration time of the pulmonary valve.

In the parasternal short-axis view, at the level of the heart base, the application of color Doppler can identify a regurgitant jet starting from the pulmonary valve. The application of continuous Doppler imaging will thus allow the calculation of the maximum regurgitation velocity and of the gradient between the pulmonary artery and the RV. This gradient, added to the RAP, will result in the estimate of MPAP.^(67,68)^ However, this measure will be feasible only in approximately 25% of situations involving critically ill patients.^(64)^

In the same section, the acceleration time of the pulmonary valve, defined as the time required for the RV outflow tract flow to reach its maximum velocity, can be obtained by applying pulsed Doppler imaging immediately proximal to the pulmonary valve. The shorter the acceleration time, the higher the pulmonary artery pressure. A value above 130 milliseconds will be considered normal, while a value below 105 milliseconds suggests pulmonary hypertension.^(69,70)^ The MPAP can be estimated using the formula 90 - (0.62 × acceleration time). Changes in heart rate may limit the accuracy of this measurement, although for MPAP values above 25mmHg, accuracy seems to be maintained.^(71)^ The identification of a systolic notch in the ejection flow indicates an increase in pulmonary vascular resistance and suggests the possibility of a precapillary mechanism.^(72)^

The acceleration time is a measure that depends on RV preload, contractility, pulmonary vascular resistance, and the intricate mechanisms between these factors. The reproducibility of acceleration time in critically ill patients is, therefore, limited to specific studies with unsatisfactory performance.^(64)^ In the transthoracic approach of a patient under MV, the correct alignment with the RV outflow tract may be problematic, and the transesophageal approach may constitute a viable alternative.

Evaluating the tricuspid regurgitant jet, Aduen et al. ^(73)^ proposed an additional method for estimating MPAP using regurgitant jet planimetry. The resulting mean gradient is simply added to the RAP, yielding an estimate of MPAP with approximately 80% accuracy against measurements obtained by pulmonary artery catheter.^(74)^ This method was later reproduced by Laver et al.^(75)^ in a population of 53 critically ill patients undergoing pulmonary artery catheterization. Although the mean difference between the MPAP measurements was only 1.9mmHg, jet planimetry for application of this technique could be obtained in only 43% of the patients, limiting its applicability.

The members of the committee did not reach a consensus about the estimation of right chamber pressures by means of bedside echocardiography by a nonechocardiographer physician. On the one hand, there is recognition that these parameters have long been used in clinical practice and are directly related to the physiology of critically ill patients and even to the calculation of traditional hemodynamic variables (e.g., pulmonary vascular resistance). On the other hand, there are uncertainties about their accuracy in the specific scenarios of emergency and intensive care and the lack of validation of many of these findings on these parameters in unstable patients. In addition, factors such as insufficient echocardiographic windows, frequent use of IMV, and the need for advanced skills on the part of the examiner to perform different quantitative measures limit the applicability of these measures in a comprehensive manner.

#### Domain 3 - Diagnostic evaluation of shock

**14. Bedside echocardiography should be routinely used in the initial evaluation of shock - 100% agreement.**

**15. Bedside echocardiography should be routinely used in the follow-up of shock and in the reassessment after institution of therapies - 100% agreement.**

**16. Bedside echocardiography contributes to the recognition of severe hypovolemia as the cause of shock - 100% agreement.**

**17. Bedside echocardiography contributes to the recognition of *cor pulmonale* as the cause of shock - 100% agreement.**

**18. Bedside echocardiography contributes to the recognition of cardiac tamponade as the cause of shock - 100% agreement.**

**19. Bedside echocardiography contributes to the recognition of severe left ventricular dysfunction as the cause of shock - 100% agreement.**

This domain was the only one to reach a positive consensus of 100% on all six questions evaluated - all of them in the first round of responses by electronic form. The use of bedside echocardiography is useful in the study of shock and should be used in the initial evaluation to help understand the mechanisms of hemodynamic instability. Ultrasound analysis will allow the evaluation of signs of severe hypovolemia, *cor pulmonale*, severe LV dysfunction, or significant pericardial effusion, making it a tool that can potentially reduce the time to diagnosis.^(76,77)^

Hypovolemic shock is characterized by a low CO due to reduced stroke volume. Cavities with reduced dimensions and low filling pressures are visualized, and sometimes, at the end of each systole, the walls touch each other, a sign described as kissing walls or systolic obliteration sign. The IVC is usually collapsed and varies greatly in diameter in the respiratory cycle.

Right ventricular failure can occur in some critical situations, such as massive pulmonary embolism and adult respiratory distress syndrome, due to the use of high ventilatory pressures to maintain an oxygenation level compatible with life.^(46)^ The RV undergoes dilation and systolic dysfunction after these gradual increases in afterload pressures, ultimately leading to obstructive shock. If the pressure on the right side becomes greater than that on the left side, there will be a paradoxical movement of the interventricular septum to the left, in addition to increasing dilation of the right chamber. These two findings together make up what we call *cor pulmonale*. In cases of acute *cor pulmonale*, we can also observe the presence of segmental alteration of the RV walls with the presence of hypokinesia or akinesia of the lateral wall with normal contraction of the apex. In cases of shock with suspected pulmonary embolism, the combined use of venous ultrasound and right ventricular dilation on echocardiogram increases the specificity of the diagnosis of PTE.^(78)^

The presence of hypoechoic content around the heart is indicative of the accumulation of pericardial fluid. The rate of accumulation of this pericardial fluid dictates how much accumulated fluid will be required to cause circulatory collapse due to tamponade. Chronic effusions rely on pericardial compliance adjustment and can reach large effusion volumes before collapse. Acute effusions, such as hemopericardium, lead to collapse more quickly due to tamponade, and approximately 50 - 100mL of blood is enough to cause shock. The timely identification of tamponade can significantly alter the treatment of patients in shock. The RA systolic collapse, added to RV diastolic collapse, is the earliest sign. The IVC becomes turgid and unchanging. Other signs that can be identified include variation in aortic, mitral, and tricuspid flow. An inspiratory variation greater than 25% measured on pulsed Doppler ultrasound at the mitral valve level and an inspiratory variation greater than 40% at the tricuspid valve level indicate the diagnosis of pericardial tamponade. Another sign that may be present is the swinging of the heart in the midst of the fluid, called swinging heart, indicating that cardiac tamponade most likely occurs in the presence of hemodynamic instability.

The use of parameters related to LV function - notably by eye-balling - in patients with shock can quickly rule out the cardiogenic mechanism. When associated with high-output states and reduced afterload, however, LV dysfunction may remain undetected, becoming evident only after reestablishment of blood volume.^(79)^

A clinical situation that deserves mention is the dynamic obstruction of the LV outflow tract. Found in up to 20% of patients with septic shock, it is associated with high mortality in the ICU.^(80)^ This can significantly change the treatment of patients with hemodynamic instability, directing the line of treatment toward systemic vasoconstrictors and inotropic and chronotropic agents, for example, for heart rate control and maintenance of euvolemia, or even administration of volume expansion aliquots. Sometimes unknown *a priori* or even having an acute onset at the time of critical illness,^(81)^ its recognition becomes essential for the intensivist qualified in advanced-level echocardiography.

The rapid ultrasound for shock and hypotension (RUSH) protocol consists of the evaluation of fluid collections in the costophrenic sinuses and pelvis, in addition to the abdominal aorta and cardiac function itself, through parasternal, apical, and subxiphoid views.^(82)^ Bagheri-Hariri et al.,^(83)^ evaluating patients in shock in the emergency room, reported a correlation coefficient of 0.84 between the result of the RUSH protocol and the final reference diagnosis. A recent systematic review identified four original studies that evaluated the diagnostic performance of the RUSH protocol.^(84)^ The positive likelihood ratio ranged between 8.25 (for hypovolemic shock) and 40.54 for obstructive shock; the negative likelihood ratio was between 0.13 (for obstructive shock) and 0.32 (for shock of mixed etiology). In general, the protocol performed better at corroborating than excluding possible mechanisms of shock.

The use of echocardiography in the evaluation of patients in shock can significantly alter the procedures adopted. Echocardiography-guided therapy of patients in shock tends to be associated with lower fluid use and greater recognition of LV dysfunction - and, consequently, the use of inotropes.^(85,86)^ The use of echocardiography in patients with shock has even been associated with better clinical outcomes in observational studies.^(86,87)^

#### Domain 4 - Hemodynamic evaluation

The assessment of blood volume in critically ill patients is a complex task that requires an integrative and multimodal approach. The use of ultrasound in this context should be viewed in the same way: The examiner should seek different tools that, through the clinical-echocardiographic correlation, will yield the most representative information. This topic may be the one that has undergone the most changes over the past few years in relation to the assessment of blood volume status and regarding how to use ultrasound parameters to assess fluid responsiveness.

Important components of blood volume that can be evaluated are the estimate of filling pressures, both on the right side (central venous pressure) and on the left side of the heart (pulmonary artery occlusion pressure - PAOP), and the estimate of extravascular pulmonary water (EVPW).

As a rule, the assessment of blood volume status takes into account variables collectively known as static, obtained at a given time, providing data on cardiac chamber pressures that do not directly inform about the responsiveness potential to fluids^(88,89)^ and that reflect complex interactions of cardiopulmonary physiology. Examples of static variables are RAP and PAOP. Specific (dynamic) parameters should be used to assess fluid responsiveness, which will be discussed in later sections.

**20. The estimation of central venous pressure by echocardiography by a nonspecialist physician is recommended as part of the hemodynamic evaluation of critically ill patients - 81.25% agreement.**

The estimation of central venous pressure - or RAP - is part of the understanding of the volume and hemodynamic status of critically ill patients and is mainly determined by venous return and right ventricular function. As a rule, the RAP measurement should be incorporated into the clinical context not in isolation but taking into account all the rest of the hemodynamic evaluation. Among other scenarios, knowledge of the RAP value is relevant both for the hemodynamic management of the patient in shock^(90)^ and for the determination of pressures on the right side of the heart, since the RV-RA gradient is imposed on it.

The RAP can be estimated by echocardiography of the IVC, according to the phase of the respiratory cycle. Because it is a highly compliant, collapsible, and contiguous vessel, the IVC directly reflects changes in the volume and filling pressure of the RA.^(91)^ Furthermore, the mechanics of the IVC remain unchanged by compensatory responses to a loss of circulating volume or the infusion of vasoconstrictors.^(92)^

The diameter of the IVC should be measured with the patient in the supine position, through a four-chamber subcostal view, from its longitudinal view, at a distance of 0.5 to 2cm from its insertion in the RA, taking care to maintain the most perpendicular alignment possible with the walls of the IVC to obtain the most faithful measurement. Measurements in the right or left lateral decubitus position can significantly change the diameter of the IVC.^(93)^ Some authors evaluated the indexation of the IVC diameter to the body surface, with inconsistent results.^(94-99)^ The interobserver correlation of IVC diameter ranges between 0.56 and 0.81 and tends to be more precise as the examiner accumulates experience.^(99-101)^

The precise method used to measure the IVC diameter has varied considerably between the studies that has evaluated the performance of this technique. While some authors sought to relate the IVC diastolic diameter with RAP,^(93-95,102-104)^ others evaluated the so-called collapse index (maximum diameter - minimum diameter/maximum diameter).^(91,105,106)^ The correlation coefficients (r) reported between RAP and diastolic diameter are between 0.72 and 0.86; between RAP. and the collapsibility index, they are between 0.57 and 0.76. Stawicki et al.^(107)^ reported an negative correlation between a 3.3% variation in the collapsibility index and 1mmHg in RAP.

The accuracy of these parameters for predicting the specific RAP value, however, is limited ^(97,105,106,108)^ due to the significant overlap of patients with normal and elevated RAP and dilated IVC, as well as the limited ability of the IVC to dilate in response to RAP increases. The identification of dilated IVC may suggest high RAP but cannot identify the magnitude of this increase.^(109)^ Extreme values of IVC diameter, however, may be useful in selected situations. When lower than 12mm, they are correlated with RAP lower than 10mmHg in patients under IMV,^(103)^ with high specificity, albeit at the expense of low sensitivity.

A number of clinical situations can result in IVC dilation without associated elevation of RAP. Athletes^(110)^ or patients with a large body surface area may similarly have spurious dilation of the IVC. In addition, portal or intra-abdominal hypertension of another nature, such as from asthma or exacerbated COPD,^(111)^ may limit our ability to properly evaluate the behavior of the IVC.

Notably, patients under IMV may have a dilated IVC only as a result of positive intrathoracic pressure. The correlation between IVC diameter and RAP was greater in spontaneously ventilated patients (r = 0.97) than in mechanically ventilated patients (r = 0.59).^(108)^ Therefore, the RAP estimate by means of IVC analysis should be primarily used in spontaneously ventilated patients (negative inspiratory intrathoracic pressure). In this population, Dipti et al.,^(112)^ in a meta-analysis of five studies conducted in the emergency room, reported that the maximum IVC diameter is consistently smaller in hypovolemic patients than in euvolemic patients. In dyspneic patients in the emergency room, the analysis of the diameter of the IVC was the most accurate ultrasound measurement for the identification of the cardiac etiology.^(113)^

The guidelines of the American Society of Echocardiography propose that by integrating the degree of inspiratory collapse and its diameter, a certain RAP value can be assigned. The degree of IVC collapse should be expressed as a percentage and as a dichotomous variable (less than or greater than 50%). This technique will allow the arbitrary assignment of one of three predetermined values (3, 8, or 15). It is not possible through this method to determine the exact value of RAP,^(114)^ and the exact precision of this strategy is not adequately documented.

Hepatic venous flow is directly related to venous flow through the atrio-caval system, thus sharing much of its behavior in different hemodynamic situations. The left and right hepatic veins drain into the IVC at the level of the diaphragm and can be evaluated by means of a four-chamber subcostal view.

The evaluation of hepatic venous flow can be used as a complementary tool in the estimation of RAP. In conditions of low or intermediate RAP, there will be a predominance of systolic flow in the liver (the systolic wave velocity - Vs - will be higher than the diastolic wave velocity - Vd). When RAP increases, systolic predominance is lost, and the Vs/Vd ratio will be less than 1. Similarly, the systolic filling fraction of the hepatic vein (systolic VTI/systolic VTI + diastolic VTI) can be calculated. A value lower than 55% is correlated with a RAP above 8mmHg with 86% sensitivity and 90% specificity.^(115)^ Although studied mainly in MV patients (unlike the evaluation of the IVC), this technique requires greater expertise on the part of the operator to obtain the appropriate window and apply Doppler imaging.

The evaluation of jugular vein dynamics through different techniques has been proposed to estimate RAP, with conflicting results.^(116-119)^ Several other techniques have been described for the evaluation of RAP, but in the understanding of this group, they are beyond the scope of the nonechocardiographer.^(109,120,121)^

**21. The estimation of left atrial pressure (LAP) by means of echocardiography by a nonspecialist physician should be part of the hemodynamic evaluation of critically ill patients - without consensus.**

PAOP is a hemodynamic parameter related to LV filling and therefore to LV diastolic function and LAP. It can be measured through cardiac catheterization or, more commonly in clinical practice, through the insertion of a pulmonary artery catheter and the occlusion of a main branch of the pulmonary artery by insufflation of its distal cuff. Echocardiography is a noninvasive alternative for the evaluation of PAOP because several echocardiographic parameters related to ventricular diastole can be used for its estimation. Among the relevant parameters, the most frequently used are the E wave, the E/A ratio, the e’ wave and the E/e’ ratio.

The E wave corresponds to the first phase of ventricular diastole (rapid ventricular filling - early filling), a consequence of the pressure gradient generated between the atrium and the LV, from the isovolumetric relaxation of the LV. In this phase of the cardiac cycle, approximately 60 - 65% of diastolic filling occurs. The peak E-wave velocity is measured by placing the pulsed Doppler sample volume immediately above the opening of the mitral leaflets in the apical four-chamber view. Under physiological conditions, the expected value of the E wave is 80 - 100cm/s. In healthy individuals, the E wave measurement alone may be a predictor of PAOP.^(122)^

After equalization of the pressure gradient between the LA and LV, the remainder of the LV filling will occur by atrial contraction, represented on transmitral Doppler as the A wave. The E/A ratio, under physiological conditions, therefore remains above 1. In situations in which LV relaxation is compromised, the LA-LV pressure gradient becomes narrower, lowering the amplitude of the E wave (E/A less than 1). In clinical situations in which there is a consequent compensatory increase in LAP, this pattern will reverse, returning E/A to greater than 1 (pseudonormal pattern) or even to greater than 2 (restrictive pattern). Nagueh et al.,^(123)^ in a population of critically ill patients, identified a significant correlation (r = 0.75) between the E/A value and the PAOP measured by pulmonary artery catheterization. Boussuges et al.^(124)^ evaluated E/A in mechanically ventilated patients, among other hemodynamic parameters, and found a positive predictive value of 100% for LAP above 18mmHg when E/A was greater than 2.

**Figure 10:**
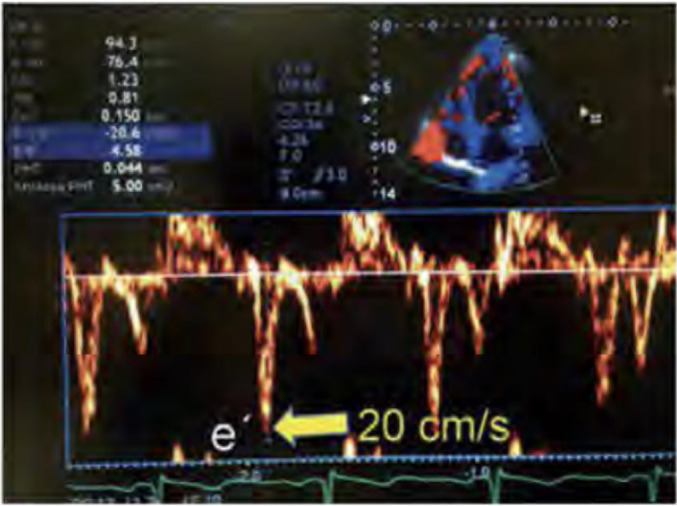
Tissue Doppler ultrasound of the basal lateral wall of the left ventricle. Note the E’ wave below the baseline during diastole (E’ or e’ wave).

Source: adapted from Barros DS, Bravim B. *Ecografia em terapia intensiva e na medicina de urgência*. São Paulo: Atheneu; 2018.

The most studied parameter for the evaluation of left diastolic pressures might be E/e’, which is an indexing of the E wave by its tissue equivalent (e’), a variable that is less subject to preload variations (Figure 10).^(125)^ Ommen et al.,^(126)^ using invasive hemodynamic parameters as a reference in patients referred for cardiac catheterization, found that the accuracy of E/e’ was 76% in relation to LV diastolic pressure, with even better results when using the septal mitral annulus lateral (or even the average between these point measurements) to measure the velocity of myocardial tissue displacement. Applying a bimodal analysis, the authors reported that 23 of 27 patients with E/e’ lower than 8 had normal diastolic pressures; similarly, all patients with E/e’ above 15 had high diastolic pressures.

These results were obtained in hemodynamically stable patients, so their generalizability to critically ill patients remains a matter of doubt. Sharifov et al.,^(127)^ through a systematic review, indicated that there is not enough evidence to properly evaluate the correlation of E/e’ with changes in LV diastolic pressure in response to exercise or pharmacological interventions, further increasing the uncertainty regarding patient instability. Also noteworthy are the frequent technical limitations related to the measurement of the e’ parameter: pathologies that affect the structure of the mitral annulus, severe mitral regurgitation, ventricular dyssynchrony, and regional contractile abnormalities. Although specific studies suggest the accuracy of this measurement even in patients undergoing MV^(128)^ and in septic shock,^(129)^ reference values have not yet been adequately validated in the population of critically ill patients.

The positive and negative predictive value of E/e’ greater than 14 were only moderate (56 and 62%, respectively) in a recent cross-sectional study that compared echocardiographic parameters with invasive measurements.^(130)^ Likewise, a recent meta-analysis of studies in patients with preserved LV systolic function^(131)^ evaluated the correlation of invasive measurements with echocardiographic parameters of diastolic dysfunction. The best accuracy was found with E/e’, although with wide variability (r = 0.19 - 0.84) and predominantly moderate correlation. The studies were underpowered (nine studies, including 286 patients, an average of 31 patients per study) and included mostly outpatients and hemodynamically stable patients.

Although these measurements are frequently taken in clinical practice and are relatively simple to obtain, taking into account the still inconsistent findings regarding the use of these parameters in critically ill patients, there was no consensus on their use. Aside from the limitations of these parameters for measuring filling pressures in critically ill patients, the prognostic power of the assessment of diastolic function has gotten attention.^(132)^ Furthermore, the combined use of diastolic function assessment with pulmonary ultrasound^(133,134)^ may provide more consistent information about the underlying mechanism in scenarios of acute respiratory failure.

**22. The estimation of EVPW by means of chest ultrasound by a nonspecialist physician should be part of the hemodynamic evaluation of critically ill patients - 87.5% agreement.**

In situations of hemodynamic instability, the decision to administer aliquots of expander solutions may be indicated, although the aggressiveness of this strategy has been a matter of debate. The increase in pulmonary capillary permeability in critically ill patients, however, can result in fluid leakage into the extravascular compartment and a consequent increase in EVPW and hypoxemia, further complicating the daily decision-making process regarding volume expansion in the ICU.

Chest X-ray continues to be used for EVPW monitoring, although its accuracy for this purpose is not ideal.^(135-137)^ Transpulmonary thermodilution is the method of choice for clinical evaluation of the amount of EVPW, although it requires the use of specialized and invasive equipment, limiting its availability at the bedside in selected settings. Through thermodilution, the expected values of EVPW are between 3 and 7mL/kg of ideal weight, while values above 10mL/kg are characteristic of pulmonary edema.^(138)^

In this scenario, chest ultrasonography is an option because the presence of enough EVPW provides enough acoustic impedance for the propagation of the ultrasonic beams, triggering the formation of artifacts known as B lines.^(139)^ The increase in EVPW is linearly correlated with the increase in the amount of pulmonary B lines.^(140,141)^ The amount of EVPW estimated by ultrasound is correlated with a worse prognosis in patients with ARDS;^(141)^ values above 14mL/kg are associated with higher mortality when detected on ICU admission.^(142)^

Volpicelli et al.^(143)^ analyzed 73 critically ill patients regarding the correlation between the pulmonary sonographic pattern (pattern A or pattern B, according to the predominance of artifacts found) and the PAOP and EVPW levels. Although the accuracy of pulmonary sonographic pattern A for the prediction of PAOP < 18mmHg was limited (sensitivity of 85.7% and specificity of 40%), the results for EVPW were promising (sensitivity of 81% and specificity of 90.9% for PLE < 10mL/kg). These findings are in agreement with previous findings,^(133)^ possibly reflecting the complexity of hemodynamic phenomena in the context of critical illness.

The dynamics of identification of B lines reflect both their precocity and fugacity. When there are significant variations in blood volume^(144-146)^ and when interpreted in the appropriate clinical setting, this finding may reflect real-time fluctuations in blood volume status. The dynamism of the findings may make it feasible to use lung ultrasound to monitor EVPW in the context of trauma or in the perioperative period of major thoracic surgery.^(147-149)^

Extravascular pulmonary water volume can be estimated by means of the quantification of pulmonary B lines using one of several protocols available.^(143,150)^ The use of simplified protocols^(137)^ is related to comparable diagnostic accuracy, even using fewer measurements.

Although many studies have evaluated the correlation between the number of B lines and both the development of clinical pulmonary edema and the direct increase in EVPW, it must be kept in mind that these were small studies (19 - 73 patients) and that it is still uncertain what is the most appropriate technique for monitoring the number of B lines and how to deal with the subjectivity in the quantification of this artifact in the eyes of the operator. Corradi et al.^(151)^ proposed the automation of this quantification by dedicated software, although these findings still lack validation in different populations. The low specificity of B lines should be taken into account in relation to the presence of previous parenchymal diseases (pulmonary fibrosis, interstitial pneumonitis), which may limit the use of this tool in an unselected population of individuals.

**23. The use of B lines in lung ultrasound can be used as a safety measure for the provision of fluids - 81.25% agreement.**

Based on the rationale of the relationship between EVPW and the increase in pulmonary B lines, some authors^(152)^ suggest that the supply of fluids, when necessary, should be guided by lung ultrasound up to the point at which the patient begins to develop B lines, indicating that the inflection point of the Frank-Starling curve has been reached. From that point on, additional fluids would only have deleterious effects.

In a study of experimental models of ARDS, Gargani et al.^(144)^ demonstrated that the appearance of pulmonary B lines occurs early in the induction of lung injury after administration of oleic acid, with concomitant worsening of compliance, but much earlier than the onset of hypoxemia. Caltabeloti et al.^(146)^ evaluated 32 patients with sepsis and ARDS and reported that the B-line ultrasound score increased by 23% when measured 40 minutes after administration of a 1,000mL aliquot of crystalloid in relation to the baseline. In contrast, the relationship between the partial pressure of oxygen and the fraction of inspired oxygen (PaO_2_/FiO_2_) remained stable at this point, suggesting that the findings by Gargani et al.^(144)^ may be mirrored in clinical studies involving critically ill patients. Theerawit et al.,^(153)^ in a study that included 20 patients admitted to the ICU, reported that the B-line ultrasound score was correlated with the increase in water balance 48 hours after admission.

In a study that evaluated 47 patients with septic shock in the emergency room, Coen et al.^(154)^ applied a structured volume expansion protocol using ultrasound parameters to replace the classic hemodynamic variables used by Rivers et al.^(155)^ B lines appeared in nine patients, warranting additional investigation of echocardiography and administration of inotropes or vasoconstrictors. However, there was no control group or differentiation between the characteristics of patients who developed and did not develop B lines. Furthermore, the mean amount of fluid administered was greater than 5L in the first 6 hours of treatment, limiting the external applicability of these findings.

Fluid responsiveness is evaluated based on the use of hemodynamic tests collectively called “functional”^(156,157)^ or simply dynamic parameters. These are maneuvers that affect cardiac function and/or the heart-lung interaction, resulting in hemodynamic disturbances. The maneuvers may consist of postural changes, respiratory cycle phases, or even infusion of small aliquots. The magnitude of the resulting hemodynamic disturbance will determine whether the individual has a greater or lesser chance of responding to fluids by increasing their CO.

Fluid administration should follow the rationale of other pharmacological interventions for critically ill patients, respecting the established indication, presentation, and dosage.^(158)^ Numerous studies have associated unfavorable outcomes both to administration of too little (with consequent impairment of tissue perfusion) and too much administration of fluids,^(159,160)^ leading to weight gain, fluid overload, and several deleterious effects in different systems.

Under the most commonly used definitions of fluid responsiveness (increase in CO of approximately 10 - 15% after rapid infusion of a 500mL aliquot of fluid), it is estimated that the proportion of fluid responders in emergency rooms and ICUs is not greater than 50%.^(161-163)^ For these reasons, the search for the answer to whether a particular patient benefits from an additional supply of fluids is one of the main issues in the routine care of the critically ill patient.

The use of echocardiographic variables may noninvasively provide information on the potential benefit of offering fluids through various parameters. These measurements can be repeated as many times as necessary to reassess the patient’s behavior over time, with variations in the clinical context, and after any interventions are performed.

**24. Inferior vena cava variability should be used as a tool for assessing fluid responsiveness - without consensus.**

The IVC is a compliant vessel, with its caliber altered by volume status, right ventricular function, and respiratory cycle. The behavior of the IVC will differ according to the patient’s ventilation - in positive pressure, it will be controlled, while under negative pressure, it will be spontaneous. The positive pressure applied to the airway in the inspiratory phase of MV will determine the engorgement of the intrahepatic portion of the IVC, which is reversed in the exhalation phase. In spontaneous ventilation, the reverse phenomenon will be observed (inspiratory collapse). The greater the impact of pressure changes in the airways on the IVC, the greater the potential for fluid responsiveness.

The transverse diameter of the IVC should be measured in the longitudinal view, through the subcostal window, caudal to the course of the suprahepatic vein. The suggested distance for a better approach to the IVC diameter is approximately 0.5-2 cm from the atrio-vena cava junction. The M mode is commonly used to facilitate the measurement process.

For patients breathing spontaneously, the most frequently used index is the collapsibility index: (maximum diameter - minimum diameter/maximum diameter × 100%).^(164)^ In patients on MV, the most common calculation method is the distensibility index: (maximum diameter - minimum diameter/minimum diameter × 100%,^(165)^ with an ideal cutoff point originally set at 18%. Feissel et al.^(166)^ used a third method of calculation, which they called the variability index: (maximum diameter - minimum diameter)/mean diameter × 100%, whose ideal cutoff point would be 12%. The qualitative assessment of IVC distensibility is an alternative to the quantitative approach and was the subject of the study by Duwat et al. ^(167)^ In those patients situated in the extremes of distensibility (< 15 and > 30%), the accuracy of the qualitative evaluation was similar to the quantitative one. In the distensibility range between 15 and 30%, however, the error rate of the qualitative evaluation reached 35%.

It is important to pay attention to the ventilatory parameters in those patients on MV. Si et al.^(168)^ reported that the diagnostic accuracy of IVC distensibility is higher in ventilated patients with a TV of > 8mL/kg predicted weight or PEEP below 5cmH2O. Similarly, almost all of the published studies included patients in sinus rhythm. Bortolotti et al.^(169)^ published the only study to date that exclusively evaluated patients with arrhythmia (53% in atrial fibrillation), reporting an area under the receiver operating characteristic (ROC) curve of 0.93 for the collapse index. Barbier and Feissel published their results independently but concurrently,^(165,166)^ both evaluating patients undergoing IMV, reporting sensitivity of 96 - 90% and specificity of 75 - 90%, respectively. Several other studies are available in this context, most of them single-center and with highly selected and limited samples (n = 15 to 90).

In the largest study to date evaluating the behavior of the IVC,^(170)^ IVC distensibility had only moderate accuracy in predicting fluid responsiveness, with low sensitivity. The authors also evaluated the end-expiratory diameter of the IVC; when evaluated at its extremes, it had a specificity of 80% for < 13mm (responders) and > 25mm (nonresponders). However, patients in these situations made up only 30% of the study population.

Several meta-analyses^(171-173)^ were performed to evaluate the aggregate performance of IVC variability for fluid responsiveness prediction. The reported sensitivity and specificity are between 63 - 76% and 73 - 86%, respectively. This diagnostic accuracy refers to a heterogeneous group of patients, including individuals under MV and spontaneous ventilation, although their physiology is different. Muller and Airapetian,^(164,174)^ evaluating only spontaneously breathing patients, reported that a collapsibility value of approximately 40% is associated with fluid responsiveness with good specificity but poor sensitivity. Préau et al.,^(175)^ through rigorous standardization of the inspiratory effort maneuver, obtained a sensitivity of 84% and specificity of 90% for a cutoff point of 48%. The application of a similar maneuver in a population of dyspneic or confused patients represents a significant obstacle to the external validity of these results. Das et al.^(163)^ conducted a recent systematic review and reported the diagnostic accuracy separately according to the ventilation modality. Among mechanically ventilated patients, the pooled sensitivity was 79%, and the specificity was 70%, resulting in an area under the ROC curve of 0.75 (13 studies; 431 individuals). In those patients on spontaneous ventilation, they identified a sensitivity of 80% and specificity of 79%, with an area under the ROC curve of 0.857 (7 studies; n = 330). The measurement of IVC variability in the spontaneously ventilated patient population agrees with previous meta-analyses^(171,172)^ but should be interpreted with caution. The ideal cutoff point varied considerably in the articles reviewed by Das et al.;^(163)^ excluding two outlier studies in each group, a trend was identified for a higher cutoff point in patients on spontaneous ventilation: 31 to 50% compared to 12 to 22% for mechanically ventilated patients.

In a study of 67 mechanically ventilated patients, Yao et al.^(176)^ recently described the distensibility index using the IVC cross-sectional area and diameter ratio, reporting areas under the ROC curve of 0.749 and 0.829, respectively. These data still lack the validation needed for greater applicability.

The evaluation of the IVC is subject to a number of technical limitations, including an adequate window, movement artifacts, and large respiratory incursions.^(177)^ Situations related to changes in central venous pressure and therefore in IVC variability should be ruled out to make the data more reliable. Among these variables, the presence of RV infarction, RV overload, or even ventilatory changes associated with the mechanical ventilator (PEEP or reduced tidal volume, for example) or with the patient himself (severe inspiratory effort) stand out.^(111)^ Furthermore, patients ventilated using methods such as pressure support or patients with intra-abdominal hypertension are not well suited to the regular use of this tool.^(178,179)^ We believe these reasons explain the lack of consensus among the committee members despite its wide use in clinical practice.

**25. Functional hemodynamic tests (minibolus and end-expiratory occlusion test (EOT)) should be used as a tool for assessing fluid responsiveness - without consensus.**

The EOT is based on heart-lung interactions and changes in respiratory dynamics that alter CO.^(180)^ The maneuver consists of performing 12 to 15 seconds of occlusion at the end of expiration. Hemodynamic measurements (including measurement of stroke volume or its correlates) should be performed before and at the end (in the last seconds) of the maneuver. The expiratory pause will induce an increase in venous return and therefore an increase in stroke volume in fluid-responsive patients.^(180-182)^ This maneuver was first described by Monnet et al.^(181)^ in a study that evaluated 34 patients on positive-pressure MV using transpulmonary thermodilution for CO measurement. It had an accuracy of 97% for the prediction of fluid responsiveness, even in patients with arrhythmia or with moderate spontaneous respiratory activity.

A recent meta-analysis^(180)^ included studies that evaluated the performance of “alternative” functional hemodynamic tests (not the traditional ones of variation in pulse pressure, variation in stroke volume, and passive leg elevation) for predicting fluid responsiveness. The EOT had an aggregate sensitivity of 86%, specificity of 91%, and area under the curve of 96%, with a positivity threshold of 5% for increased stroke volume or its substitutes. The exclusion criteria varied between the studies, but it is noteworthy that the exclusion was due to an unsatisfactory echocardiographic window, spontaneous breathing during the test, complex arrhythmias (ventricular tachycardia), and *cor pulmonale*.^(181,183)^ The methods for measuring CO were varied, with transpulmonary thermodilution predominating.

Two recent studies evaluated whether the measurement of VTI by echocardiography can serve as a response variable to EOT. Jozwiak et al.^(183)^ evaluated 30 patients under positive-pressure MV and reported that the accuracy of the maneuver was 93.8% with a cutoff point of 5% in the VTI increment. Georges et al.^(184)^ evaluated 50 neurocritical patients and found a 9% increase in VTI as the ideal cutoff point, with a sensitivity of 89% and specificity of 95% (area under the ROC curve 96%).

The EOT may be appropriate in different clinical scenarios, especially when the passive leg lift test is not applicable, such as when there is intra-abdominal or intracranial hypertension or traumatic fracture of the hip or leg.^(180)^

Perhaps the functional test closest to a conventional fluid challenge with a simpler mechanism is the so-called minibolus test, in which a small aliquot is administered to the patient in question, and the hemodynamic effects of this intervention are monitored in real time. Regarding the other functional tests, the minibolus test was initially proposed to use echocardiography as the method of response measurement. In its original description,^(185)^ after the administration of 100mL of colloid solution in 1 minute, each 10% increase in VTI had a specificity of 78% and sensitivity of 95% at discriminating responders from nonresponders. Along the same lines, Wu et al.^(186)^ used an even smaller infusion volume (50mL) and crystalloid solution. These authors reported lower sensitivity and higher specificity than the previous study. Other authors have validated the minibolus technique in other contexts, using other methods to measure CO,^(187-189)^ predominantly pulse contour analysis and transpulmonary thermodilution,^(180)^ with similar diagnostic performance.

Aspects such as the need for high precision on the part of the examiner to identify differences of the order of 5 to 10% (which could be related to variation inherent in the method, for example), as well as the lack of reproducibility of studies in larger populations of critically ill patients, may explain why there was no consensus on the regular use of functional hemodynamic tests to predict fluid responsiveness.

**26. The passive leg elevation maneuver should be used as a tool for assessing fluid responsiveness - 93.75% agreement.**

Elevation of the legs in response to hypotension has been empirically employed in different contexts.^(190-192)^ Its goal is to drain blood held in the venous system of the leg to the RA, thus optimizing venous return and, consequently, CO. Approximately 300mL of blood^(193-195)^ will be mobilized through gravitational transfer, which constitutes an endogenous - and reversible - volume challenge, countering the effects of water overload and its deleterious consequences in the most diverse of contexts.^(89)^ If the ventricles are operating in the Frank-Starling preload-dependent region, CO will transiently increase, most evidently approximately 60 to 90 seconds after the maneuver.^(196)^ Thus, an essential component of the maneuver is to verify its effect on CO in real time. The ideal tool for this purpose should allow the detection of quick variations in CO, ideally in a continuous manner. Echocardiographic evaluation, although essentially intermittent, has been evaluated as an alternative in this context, with consistent results.^(197-201)^ Wrist contour analysis has become one of the most commonly used tools to verify the response to leg elevation. When compared to pulse contour analysis or esophageal Doppler examinations, for example,^(202,203)^ transthoracic echocardiography has similar performance.

Two meta-analyses of more than 20 studies, comprising approximately 1,000 patients, evaluated the performance of passive leg raising as a predictor of fluid responsiveness.^(202,203)^ The reported sensitivity and specificity were 0.85 - 0.86 and 0.91-0.92, respectively, with an area under the ROC curve of 0.95 in both studies and an ideal cutoff point of 10%.^(203)^ The diagnostic accuracy was similar regardless of the initial position (supine or elevated headboard) and whether the individual was on spontaneous or controlled ventilation.^(202)^

Although most studies have been conducted in patients with regular rhythm, Kim et al.^(204)^ evaluated only patients with atrial fibrillation in the postoperative period of cardiac surgery and reported an accuracy of up to 77% for predicting fluid responsiveness, although thermodilution was used as a tool for monitoring CO variations. The use of alternative ultrasound parameters to evaluate the response to the maneuver has been described, with similar results using femoral^(200)^ or carotid Doppler ultrasound.^(205)^ These are viable options in case of difficulty in obtaining aortic outflow tract flow measurement.

The use of echocardiography as the response variable of the maneuver by means of the VTI measurement has the fundamental limitation of obtaining an adequate window and angle in a timely manner. Also noteworthy is intra-abdominal hypertension; compression of the IVC may limit the drainage of fluid from the lower limbs to the RA, resulting in compromised test accuracy due to false-negatives.^(206,207)^ In addition to these aspects, severe hypoxemia, high risk of aspiration of gastric contents, and intracranial hypertension should prompt caution in the application of the maneuver.

**27. The estimation of CO from VTI measurement should be used as a tool for hemodynamic evaluation - 100% agreement.**

The estimate of CO will be relevant in situations in which there is diagnostic doubt about the mechanisms of hemodynamic deterioration or when intervening in CO is considered, such as with inotropic drugs. Echocardiography is the first option for discerning the mechanism of shock, as well as for its evaluation.^(208,209)^ The product of the VTI and the area of the LV outflow tract equals the stroke volume, which, multiplied by the heart rate, equals CO.^(210)^

Dinh et al.^(211)^ evaluated the accuracy of emergency physicians with limited and focused echocardiographic training to obtain the VTI measurement in determining the CO of 100 emergency room patients. In all patients, it was possible to measure the LVOT diameter, although in three individuals it was not possible to measure the VTI. When validated by a cardiologist, the LVOT diameter measurements were optimal in 90% of the cases. Regarding the VTI measurements, 78% were classified as such (numbers similar to those obtained by certified echocardiographers). The mean difference in VTI measurement between emergency physicians and echocardiographers was 8%, with a Pearson’s correlation coefficient of 0.87.

Echocardiography has some advantages over continuous invasive methods: It is noninvasive; has lower cost; is not influenced by hypothermia; allows morphological evaluation of the heart, with analysis of valves, chamber, and pericardium size; allows the quantification of global and segmental functionality; and can be integrated, for example, with lung ultrasound.

Several aspects may limit the accuracy of echocardiographic measurement, especially due to visualization limitations thar arise from too-small cardiac windows and deviation of the alignment of the Doppler interrogation axis from the real blood flow. The presence of pathologies that affect the aortic valve - both stenosis and regurgitation - interfere with the accuracy and often make measurement impossible. Atrial fibrillation requires taking several VTI measurements to obtain a reliable mean value, due to the variability of the measurements from heartbeat to heartbeat.^(212)^

Most studies that have evaluated the agreement of CO estimation by echocardiography with intermittent thermodilution have used transesophageal echocardiography in patients in the perioperative period of cardiac surgery, in conditions of hemodynamic stability and IMV.^(213)^ The patient populations have consisted mostly of individuals in sinus rhythm, without significant valvular pathologies. Crossingham et al.,^(214)^ in a recent systematic review, reported marginal to acceptable agreement between echocardiography and conventional thermodilution using a pulmonary artery catheter. transpulmonary bypass, and pulse contour analysis, among other tools. Mercado et al.^(215)^ recently reviewed the agreement between intermittent thermodilution and echocardiography. In a study that included 38 mechanically ventilated, sedated patients in sinus rhythm, the authors verified the accuracy and precision of echocardiography for estimating CO, with narrow deviation and acceptable limits of agreement, in addition to its good ability to detect trends. In that study, the variation in CO estimated by echocardiography had a sensitivity of 88% and specificity of 66% to detect a 10% variation in CO measured by thermodilution.

## CONCLUSIONS

The purpose of this document is to synthesize information and discuss points of interest that may improve the performance of bedside echocardiography by physicians who are not specialists in echocardiography. Using the Delphi method, participants from medical associations representing different practice areas responsible for the care of critically ill patients reached consensus on most questions pertinent to the use of bedside echocardiography by physicians who are not specialists in echocardiography.

The positions described in this document reflect the goals of bedside ultrasound by nonspecialist physicians and prioritize direct qualitative parameters that may affect decision-making. Essentially quantitative parameters that require strictly precise measurements or lack validation in the literature in critically ill patients engendered rejection or even lack of consensus among the committee members. Furthermore, there was a particular trend in the ability to reach consensus in relation to each of the domains addressed. The domain related to the assessment of shock enjoyed consensus on all questions from the beginning of the process, while domains such as assessment of left ventricular systolic function and hemodynamic assessment concentrated questions that remained without consensus at the end of the process.

Consensus documents are not guidelines and have the ultimate goal of creating opportunities for improving the quality of care in a given area. They are based on the opinion of experts and are primarily informative and educational. The issues addressed throughout this text may reflect uncertainties and be influenced by personal points of view. The rigorous method used to obtain this consensus aims to mitigate personal biases and identify the position of a group of people dedicated to the optimization of bedside echocardiography.

## Appendix 1 PubMed/Medline^®^ search strategy

### Domain 1: Assessment of left ventricular function

((“Echocardiography”[Mesh] AND [“Intensive Care Units”[Mesh] OR “Emergency Medical Services”[Mesh] OR “Hospital Medicine”[Mesh]])) AND ((“Heart Ventricles”[Mesh] OR “Systole”[Mesh] OR “Ventricular Dysfunction, Left”[Mesh] OR “Heart Failure, Systolic”[Mesh])) AND ((Spanish[lang] OR Portuguese[lang] OR English[lang] ) AND adult[MeSH]))

### Domain 2: Assessment of right ventricular function

((“Echocardiography”[Mesh] AND [“Intensive Care Units”[Mesh] OR “Emergency Medical Services”[Mesh] OR “Hospital Medicine”[Mesh]])) AND (“Heart Ventricles”[Mesh] OR “Pulmonary Heart Disease” OR “Respiratory Distress Syndrome, Adult”[Mesh] OR “Hypertension, Pulmonary”[Mesh])

### Domain 3: Hemodynamic evaluation

((“Echocardiography”[Mesh] AND [“Intensive Care Units”[Mesh] OR “Emergency Medical Services”[Mesh] OR “Hospital Medicine”[Mesh]])) AND (“Hemodynamics”[Mesh] OR “Cardiac Output”[Mesh] OR “Stroke Volume”[Mesh] OR “Vena Cava, Inferior”[Mesh] OR “Extravascular Lung Water”[Mesh] OR “Circulatory and Respiratory Physiological Phenomena”[Mesh])

### Domain 4: Diagnostic evaluation of shocks

((“Echocardiography”[Mesh] AND [“Intensive Care Units”[Mesh] OR “Emergency Medical Services”[Mesh] OR “Hospital Medicine”[Mesh]])) AND (“Shock”[Mesh] OR “Hypovolemia”[Mesh] OR “Cardiac Tamponade”[Mesh] OR “Shock, Septic”[Mesh])
